# Shiga Toxin-Associated Hemolytic Uremic Syndrome: Specificities of Adult Patients and Implications for Critical Care Management

**DOI:** 10.3390/toxins13050306

**Published:** 2021-04-26

**Authors:** Benoit Travert, Cédric Rafat, Patricia Mariani, Aurélie Cointe, Antoine Dossier, Paul Coppo, Adrien Joseph

**Affiliations:** 1 Service de Médecine Interne, Hôpital Bichat, Assistance Publique-Hôpitaux de Paris, 75018 Paris, France; btravert@hotmail.com (B.T.); antoine.dossier@aphp.fr (A.D.); 2 Centre de Référence des Microangiopathies Thrombotiques (CNR-MAT), Assistance Publique-Hôpitaux de Paris, Hôpital Saint-Antoine, 75012 Paris, France; cedric.rafat@aphp.fr (C.R.); paul.coppo@aphp.fr (P.C.); 3 Urgences Néphrologiques et Transplantation Rénale, Hôpital Tenon, Assistance Publique-Hôpitaux de Paris, 75020 Paris, France; 4 Service de Microbiologie, Hôpital Robert Debré, Assistance Publique-Hôpitaux de Paris, 75019 Paris, France; patricia.mariani@aphp.fr (P.M.); aurelie.cointe@aphp.fr (A.C.); 5 Service d’Hématologie, Hôpital Saint-Antoine, Assistance Publique-Hôpitaux de Paris, 75012 Paris, France; 6 Médecine Intensive Réanimation, Hôpital Saint Louis, Assistance Publique-Hôpitaux de Paris, 75010 Paris, France; 7 Centre de Recherche des Cordeliers, Équipe Labellisée par la Ligue Contre le Cancer, Inserm U1138, Université de Paris, Sorbonne Université, 75006 Paris, France

**Keywords:** Shiga toxin, *Escherichia coli*, hemolytic uremic syndrome, thrombotic microangiopathy

## Abstract

Shiga toxin-producing *Escherichia coli*-associated hemolytic uremic syndrome (STEC-HUS) is a form of thrombotic microangiopathy secondary to an infection by an enterohemorrhagic *E. coli*. Historically considered a pediatric disease, its presentation has been described as typical, with bloody diarrhea at the forefront. However, in adults, the clinical presentation is more diverse and makes the early diagnosis hazardous. In this review, we review the epidemiology, most important outbreaks, physiopathology, clinical presentation and prognosis of STEC-HUS, focusing on the differential features between pediatric and adult disease. We show that the clinical presentation of STEC-HUS in adults is far from typical and marked by the prevalence of neurological symptoms and a poorer prognosis. Of note, we highlight knowledge gaps and the need for studies dedicated to adult patients. The differences between pediatric and adult patients have implications for the treatment of this disease, which remains a public health threat and lack a specific treatment.

## 1. Introduction: Overview of Thrombotic Microangiopathies in Adults

Thrombotic microangiopathy (TMA) syndromes share a common pathologic description of arterial, intra-renal or systemic micro-vascular occlusion, resulting from endothelial aggression accompanied by the formation of platelet aggregates. The definition is thus histological, and the classic triad associating peripheral thrombocytopenia, mechanical extra-corpuscular anemia, and end-organ damage of varying severity is the clinical translation of this syndrome [1].

Two distinct major syndromes of TMA were originally described: thrombotic thrombocytopenic purpura (TTP), and hemolytic uremic syndrome (HUS) [2]. TTP, whose first clinical description was reported by Elie Moschcowitz in 1925 [3], was historically defined as a form of TMA with predominant cerebral involvement, severe thrombocytopenia (typically less than 30 × 10^9^/L) and the absence of severe renal impairment. Conversely, HUS, a term coined for the first time by Conrad von Gasser in 1955 to describe a series of five pediatric cases, is characterized by predominant renal involvement, moderate thrombocytopenia, and less frequent neurological involvement [4]. However, significant overlap between the clinical and biological features deemed specific to TTP and HUS precludes any definitive etiological diagnosis based on these sole criteria. In addition, TMA syndromes far transcend the dual HUS/TTP categorization, encompassing a myriad of other causes encountered in various settings, such as infections, malignant hypertension, cancers, systemic autoimmune diseases, pregnancy, transplantation or drugs [2,5,6]. Major advances in the understanding of the pathophysiological mechanisms underpinning these different forms of TMA over the past thirty years have been a real turning point, permitting comprehensive nosologic delineation. Most of all, they have paved the way for targeted therapies, such as eculizumab in atypical HUS [7]. Hence, the diagnosis of TTP hinges on the demonstration of an acquired, immune-mediated or congenital deficit in ADAMTS13.

Historically, HUS was split between so-called “typical” HUS and “atypical” HUS, a terminology meant to reflect the fact that, in children, the description of Shiga toxin-producing *Escherichia coli* (STEC) HUS both predates and largely outnumbers (by a ratio of 10/1) the cases due to “atypical” HUS [8]. Moreover, “typical” HUS is supposed to have a distinctive and uniform clinical presentation, with bloody diarrhea at the forefront. The diagnostic work up of STEC-HUS now largely relies on the identification of the toxin and/or a pathogenic strain of *E. coli.* “Atypical HUS” typically comprises causes of HUS stemming primarily from genetic variants affecting complement regulatory proteins or less frequently due to autoantibodies directed against key regulators of the complement, mostly factor H [9]. Yet, atypical HUS also includes genetic causes of HUS remotely related, or even unconnected, to the complement: pathogenic variants of diacylglycerol kinaseε (DGKE), plasminogen or Cobalamin C deficiency represent such well-acknowledged causes of HUS [7]. Finally, secondary HUS encompasses a vast and composite group of TMA etiologies not otherwise classified in the previous categories (although the involvement of the complement is still under scrutiny in some cases) [2,7].

The global burden of STEC infection worldwide has been estimated to be around 43.1 acute illnesses per 100,000 person-years, leading to 3890 annual cases of STEC-HUS [10], the vast majority of STEC infections remaining uncomplicated. STEC-HUS occurs mostly in children and is considered exceptional in adults, apart from specific outbreaks. Despite the much lower incidence of STEC-HUS in adults compared to children, the majority of STEC-HUS related deaths occurs in people ≥60 years old [11]. In 2011, an exceptional European epidemic mainly affected Germany (3816 cases, 845 with HUS, 54 deaths), and France (24 cases in the Bordeaux region, nine patients with HUS including eight adults). It was eventually traced to an atypical hybrid pathotype O104:H4 strain combining enteroaggregative and enterohemorrhagic virulence and producing an extended spectrum beta-lactamase. This outbreak, related to organic fenugreek sprout consumption, mostly affected adults (88%, median age 42 years) and contributed to raised awareness of the risk and the severity of STEC-HUS in adults. However, series of adult STEC-HUS cases remain rare [12,13,14]. Hence, the clinical characteristics of STEC-HUS in adults, as well as the impact of therapeutic strategies on outcome, remain uncertain.

In this narrative review, we will summarize the current evidence on the epidemiology, pathophysiology, clinical presentation, diagnosis and prognosis of STEC-HUS patients, focusing on the specific features of adult cases and their implication for clinical practice.

## 2. Historical Perspective

The Shiga toxin (Stx) is a lethal toxin first isolated from Shigella dysenteriae type 1 by Gerald T. Keusch in 1975 [15]. In 1977, Jack Konowalchuk et al. isolated from several strains of *E. coli* including O26 serogroup, a protein toxic to Vero cells (a line of renal epithelial cells isolated from the African green monkey), hence its original name, Verotoxin [16]. Nowadays, both terms can be used, even though “Shiga toxin” is preferred in this review. Dr. O’Brien and colleagues went on to demonstrate that a toxin from an enteropathogenic *E. coli* shared the same structural and immunological properties with the Stx and succeeded in 1983 in purifying the toxin in an O157:H7 *E. coli* strain isolated from stool samples of patients with bloody diarrhea [17]. The same year, the toxin expression in *E. coli* was linked to a set of genes carried by a lambda bacteriophage [18]. Another major step for STEC-HUS description was achieved by Mohamed A. Karmali et al. with the identification of Stx in the stools of eight cases of sporadic HUS, further reinforcing the association between Stx and HUS [19].

## 3. Shiga Toxin Producing *E. coli*

The term Shiga toxin-producing *E. coli* refers to an *E. coli* strain that acquired the capacity to produce a Stx, through transfer of the gene by a Stx phage. The toxins produced by STEC are classified as type 1 (Stx1) and type 2 (Stx2), immunologically distinct, and several Stx1/Stx2 subtypes (Stx1a, Stx1c, Stx1d and Stx2a to Stx2g) have later been reported based on phylogenetic analysis of Stx sequences [20,21]. Hundreds of STEC serotypes have been described, based on their somatic (O) and flagellar (H) antigens, dozens of which are involved in human disease [22,23]. Most STEC harbor the locus of enterocyte effacement pathogenicity island within their genomes, including, among other pathogenic genes, an adhesin called intimin encoded by the *eae* gene and allowing for the intimate attachment of the bacteria to the intestinal epithelium [24].

The historical serogroup O157 is the predominant serogroup worldwide, but non-O157 serogroups are becoming increasingly prevalent in part due to the improved availability of molecular tools permitting their detection [25,26].

## 4. Epidemiology

### 4.1. Source of Data

Data pertaining to STEC infections in humans stem from case observations, outbreak descriptions, or clinical and microbiological surveillance networks. Prospective studies and randomized controlled trials are scarce, particularly in adults.

Surveillance networks with an active case reporting system are part of foodborne disease monitoring networks, and include FoodNet in North America, the European Center for Disease Prevention and Control (ECDC), or PulseNet, a global network dedicated to laboratory-based surveillance for foodborne diseases in 85 countries [27]. They mainly rely on pediatric centers for active surveillance, along with passive surveillance from laboratories. Thus, STEC-HUS incidence as reported in the adult population might be underestimated and should be interpreted very cautiously.

In addition, the diagnostic criteria of HUS, and the diagnosis of STEC infection vary in the literature, whether it is based on the detection of Stx by PCR [28] or on the identification of an O157:H7 strain in the stool, especially for the earlier studies before the generalization of culture-independent testing for Stx [11,29].

### 4.2. Overview of the Main STEC-HUS Outbreaks Involving Adults

In 1982, a first hemorrhagic diarrhea outbreak secondary to O157:H7 STEC was described in Michigan and Oregon, USA, causing gastrointestinal illness and bloody diarrhea, but without any HUS cases reported [30].

In 1985, in southwest Ontario, Canada, an outbreak of O157:H7 STEC-associated hemorrhagic colitis affected 55 residents and 18 staff members of a nursing home and was responsible for 12 cases of HUS, causing 17 deaths (11 with HUS) [31]. A massive STEC O157:H7 outbreak occurred in Washington between December 1992 and February 1993. Originating from contaminated hamburger meat distributed in a fast-food chain, the outbreak accounted for 501 cases of bloody diarrhea (including 125 adults), which resulted in 151 hospitalizations and 45 cases of HUS and claimed three lives [32]. The largest known STEC O157:H7 outbreak took place during the summer of 1996 in Japan. It was traced to contaminated white radish sprouts eaten in salads, affecting more than 12,000 people during several epidemic waves and causing three deaths [33]. It spread in schools in the city of Sakai in the Osaka region, overwhelmingly affecting children from 62 different elementary schools, resulting in more than 400 hospitalizations and 121 HUS cases [33,34]. In addition, 47 adult cases were recorded, including three HUS cases. All adults worked in the same factory, in the Kyoto region, and the food source was identical [35].

During winter 1996, an O157:H7 STEC outbreak occurred in Lanarkshire county, in Scotland, causing 262 cases of bloody diarrhea, mostly in adults (79%, median of age 71), including 28 HUS (11%) and sixteen deaths. Among the 28 adults with HUS, 16 patients died [36,37].

In June 1999, the Jiangsu and Anhui outbreak in China caused 195 HUS, resulting in 177 deaths. Again, mostly middle aged and elderly patients were infected as 167 (85.6%) were over 50 years old whereas only two patients were less than 20 years old. This outbreak was connected to a new STEC O157:H7 clone, phylogenetically closely related to the Sakai one. The reservoir responsible for the contamination could not be clearly identified [38].

An exceptional outbreak hit Europe in 2011. Germany bore the brunt of the outbreak with 3816 cases and 845 HUS. Eighty-eight percent of the patients were adults with a median age of 42. Fifty-four deaths were reported. The French health authorities disclosed 24 STEC infections and nine HUS, including eight adults, with no reported deaths. It disseminated via contaminated organic fenugreek sprouts and signaled the emergence of an atypical hybrid pathotype O104:H4 strain combining enteroaggregative and enterohemorrhagic virulence features as well as an extended spectrum beta-lactamase phenotype [12,13,14,39].

Since 2011, multiple outbreaks have been reported, involving mostly non-O157:H7 strains:

- O111 strain in 2011 in Japan (156 STEC infections including 65 adults and 26 HUS) [40];
- O145 strain in 2010 in Ohio and Michigan, associated with romaine lettuce consumption (27 infections, median age 19 (range 13 to 31) years, three HUS) [41];
- Seasonal outbreaks of O55:H7 strain infections in south-west England between 2014 to 2015 (31 infections including 11 adults, 13 HUS including two adults) [42];
- O121 strain infection in Canada in 2016 to 2017, associated with uncooked flour consumption (30 infections, median age 23.5 (range 2 to 79) years, one HUS) [43];
- O26:H11 strain infection in South Africa in 2017 (four HUS, all < five years) [44] and in France in 2019 from cow’s milk soft cheese consumption (16 infections including one adult, 14 HUS including no adults) [45].

To compare the risk of HUS and deaths in STEC outbreaks between children and adults, we ran a meta-analysis with a random effect model, which provided a pooled prevalence of HUS of 15% (95% CI 10–22) in adults and 17% (2–67) in children, and a proportion of death of 9% (4–17) in adults and 1% (0–10) in children. (Table 1).

### 4.3. Incidence

The incidence of STEC-associated infections is estimated on a regular basis in the USA by the FoodNet annual reports. In 2018, it stood at 62.6 per 1,000,000 person-years with an incidence of STEC-HUS of 6.0 per 1,000,000 person-years [50]. In Europe, the global incidence of STEC-HUS was assessed in Germany around 1.1 per 1,000,000 person-years in 2008 to 2012 [51], and in children in France, around 10.0 per 1,000,000 children-years in 2007 to 2016 [52]. Appraisal of STEC-HUS incidence in adults based on the Oklahoma registry data was 0.68 per 1,000,000 person-years in 1989 to 2006, which was 10-fold less than the incidence of STEC-HUS in children (6.3 per 1,000,000 children-years). The interpretation of these results is rendered dubious by the fact that only five out of 21 adults had a confirmed STEC infection, while severe ADAMTS13 deficiency was documented in two cases, suggesting a final diagnosis of TTP [53]. In adults, the incidence of both STEC infections and STEC-HUS tended to be higher in older people, compared to adults aged 18 to 59 in FoodNet reports [11,25,54].

Based on the meta-analysis of surveillance network studies reported in Table 2, the risk of developing HUS following a STEC infection is 1% (1–2) in adults and 9% (7–12) in children, while the risk of death is 1% (1–1) in adults and 0% (0–1) in children.

Geographical disparity is important, with contrasting figures even across Europe. For example, the number of STEC infections was reported to be as high as 74 per 1,000,000 person-years in the Netherlands or 50 per 1,000,000 person-years in the UK compared to 2.9 per 1,000,000 person-years in Germany [55]. Nevertheless, the epidemiology remains largely unknown in many regions [10]. The highest incidence of STEC-HUS was observed in Argentina in 2002, with 122 per 1,000,000 person-years [56]. There has been a constant increase of worldwide STEC infection incidence observed over the last decade, although these results have not been substantiated by data deriving from the USA, suggesting regional disparities [50]. More than a sustained trend, these results may also reflect a surveillance bias resulting from the increased use of culture-independent testing for Stx performed by clinical laboratories, a change in diagnostic test accuracy and clinician practice [57]. In Europe and Northern America, the vast majority of cases occur sporadically, regardless of whether STEC gastro-intestinal infections or STEC-HUS is considered [10,29,52,53,54,58,59].

### 4.4. Global Burden Estimation

To date, the meta-analysis published in 2014 by Majowicz et al. remains the only effort to estimate the global burden of STEC infections worldwide, with an estimation of 43.1 STEC infections per 100,000 person-years based on data obtained from 1990 to 2012 [10].

### 4.5. Mode of Transmission

Routes of transmission initially described in outbreaks are mostly foodborne, accounting for approximately half of outbreaks in the USA reported before 2002, whereas 14% arose from inter-individual transmission, 6% from recreational water, and 21% of outbreaks remained of unknown origin [67]. In 2014, foodborne STEC infections were deemed responsible for 61.6% of the cases, 7.1% originated from animal contact and 28.3% were related to person-to-person transmission, with no case of waterborne infections [54].

The diminishing share of waterborne infections reported in high income countries may be a result of improvement in sanitary controls which have aimed to reduce the risk of water contamination stemming from cattle and humans, but water probably remains a likely cause of transmission in emerging countries [68].

Human-to-human transmissions have been incriminated in secondary cases during outbreaks, including in Japan in 1996 and in Europe in 2011 [14,34,39] and among households [69].

The preponderance of raw beef and hamburger meat as a source of contamination seems to have declined nowadays in favor of fresh products such as leafy greens, lettuce [50], or sprouts [12,70], which are now recognized as the second most common source of foodborne transmission [71]. In comparison to O157 *E. coli*, non-O157 strains display enhanced biofilm formation attributes as well as increased adherence to green leaves, which may account for the rising role of these strains [72,73]. In France, contaminated raw milk cheese consumption was the source of a major outbreak in 2018, and this argues for the avoidance of non-pasteurized cheese in children under six years of age [45].

Overall, the diversity of transmission modes is a major contributor to geographical disparity, and local consumption habits must be taken into account when investigating the origin of an outbreak.

### 4.6. Predictors of STEC-HUS in Adults

There is an important heterogeneity of age and sex distribution of HUS among reports of patients with STEC infections, as well as stark differences with regard to mortality and the likelihood of HUS [74].

These contrasting results are rooted in various epidemiological, demographic, clinical and microbiological characteristics: the geographical area, source of contamination, place of transmission affecting communities of children, adults or the elderly, risk factors associated with patients such as age, sex and comorbidities, and microbiological characteristics of the strain. In addition, for any given isolate defined by its phylogenetic lineage, age may enhance the risk of HUS [75].

#### 4.6.1. Age

The incidence of STEC-related infections in the pediatric setting is higher than in adults. It is well known that children under five years are exposed to a heightened risk of developing STEC infections [11,29,37,50,54,56,58,60], with a peak incidence of between six months and two years [25,76]. Settings in which an outbreak occurs determine the distribution of STEC-HUS cases by age group, depending on whether the onset and spread involves nursing homes [31,46], nurseries and schools [34,42,47], or corporate catering and restaurants [30,32,35]. The risk of HUS decreases in young adults before increasing sharply in older patients. Indeed, both the global incidence of STEC infections [77] and the risk of developing HUS after STEC infection increase over 60 years of age compared to younger adults [11,31,37,54,61,78]. In surveillance network studies, children represent half of STEC infections, and the vast majority of HUS cases [11,53,63] (Table 2).

#### 4.6.2. Sex

There is a slight but consistent predominance of female patients with STEC infections across reports of sporadic cases [11,25,28,29,54,58,63,78] and outbreaks [14]. Data addressing the issue of whether female patients are at increased risk of HUS in the case of STEC infection have yielded conflicting results [11,40,77,78]. Female predominance has been tied to an overall greater environmental exposure in women and more specifically to socio-behavioral differences affecting the diet, as during the 2011 O104:H4 outbreak [14,39]. To support this hypothesis, female predominance in this outbreak decreased at the end of the outbreak, when human-to-human transmission accounted for a larger proportion of cases [14].

#### 4.6.3. Hypochlorhydria

Hypochlorhydria, whether related to gastrectomy or to proton pump inhibitors, has been associated with an increased risk of STEC infection and STEC-HUS [37,79].

#### 4.6.4. Antibiotics

The role of antibiotics in STEC infection is discussed in the treatment section. Briefly, antibiotics inducing the expression of SOS-response genes (ß-lactams, fluoroquinolones and cotrimoxazole) are associated with an increased risk of HUS in patients infected with STEC [80], whereas antibiotics with anti-infectious properties which rely on protein and cell wall synthesis inhibition (e.g., aminoglycosides, fosfomycin or azithromycin) may be protective against STEC-HUS evolution or STEC shedding [81,82,83].

### 4.7. Epidemiological Impact of Microbiological Characteristics

Non-O157 strains play a growing role in the epidemiology of sporadic cases and outbreaks of STEC, while the share of STEC O157 is decreasing. This course has followed a consistent trend in the USA over the last twenty years [25,50]. However, most of the strains have previously been described in sporadic cases and outbreaks in the USA over the period ranging from 1983 to 2002 (O26, O111, O103, O121, O45, O145) [25,26]. A few others are genuinely emerging strains and have come under scrutiny in the last decade. Some, such as STEC O80 and O91, are noteworthy for their outstanding epidemiologic and virulence profile.

A similar evolution has been observed in European countries such as Switzerland [62], Belgium [84], Norway between 1992 and 2012 [28], and France [76], whereas the strain distribution appears to have been stable in Germany between 2008 and 2012 [51].

Historically, STEC O157:H7 was regarded as the most pathogenic strain [25], with a risk of HUS around 10%, compared to around 1% for non-O157 strains [25,79,85]. However, recent outbreaks, such as the O104:H4 in 2011 [14], or compared data from surveillance networks in Switzerland [59,86] and France [65], have called this paradigm into question.

Strain distribution varies between pediatric and adult STEC-HUS, with a lower incidence of O157 in adults [65,87,88].

STEC O91 is an emerging strain expressing Stx2 and negative for *eae*, which seems prone to affect adults and is associated with an important risk of HUS [89]. It was not described in the pediatric cohort in 2009 to 2016 [76] while it was the predominant strain in the adult cohort of STEC-HUS over the same period in France [65]. Similarly, it was only described in adults in Switzerland in 2017 [62], and it was the most commonly identified O group in adults with STEC infections in Germany [88].

STEC O80 represents another virulent emerging strain in Europe, affecting both children and adults with a worryingly growing incidence [52,62,90].

The type of Stx has a major bearing on the likelihood of developing HUS during the course of an infection with STEC. Stx2, and more specifically the subtype Stx2a, has been associated with HUS [78,86,91], whereas Stx1 [28], Stx2c [28,78] and the association of Stx1 and Stx2 [92] were found to be protective against HUS, consistent with in vitro models [93].

Of note, Stx type correlates with the *E. coli* strain: for instance, STEC O157 in Europe and North America almost always expresses Stx2 (alone or in combination), and almost never exclusively expresses Stx1 [25,29,58,59,63,78].

The *eae* gene encoding intimin is associated with diarrhea, bloody diarrhea, and inconstantly with HUS [28,86].

## 5. Physiopathology

After ingestion of a contaminated meal, STEC penetration through the mucus layer and the intestinal barrier are facilitated by secretion of the StcE metalloprotease [94] and the Locus of Enterocyte Effacement [95], encoding a wide range of effector proteins common to enterohemorrhagic and enteropathogenic *E. coli* [24]. Thereafter, STEC release their Stx, which translocate across the intestinal epithelium [96,97]. How it manages to home in on target organs is still debated [98]. The receptor for Stx, globotriaosylceramide (Gb3) is expressed on human endothelial cells, podocytes [99], mesangial cells, tubular and glomerular epithelial cells and neurons [100]. Once fixed on their receptor, Stxs are internalized via endocytosis and undergo retrograde shuttling to the endoplasmic reticulum, from where the A subunit unleashes its cytotoxic effects through the inhibition of protein synthesis by the 60S ribosomal subunit. Besides their ribotoxic effect, Stxs are able to elicit the production of inflammatory cytokines by target cells [101], modulate the host immune response [102,103] and activate the complement pathway [104,105]. The clinical progression from STEC infection to TMA also involves endothelial dysfunction [106] and induction of a thrombogenic phenotype [107] via an increase of tissue factor [108], von Willebrand factor [109] and platelet activation [110,111].

The pathophysiological mechanisms responsible for the uneven case distribution across age groups are unclear. Initially suspected to decrease with age, a recent study showed that Gb3 expression appears in fact to be stable throughout life [112]. Immuno-senescence is another plausible culprit: the decrease in the rate of antibodies against Stx1 and Stx2 observed after the fifth decade in humans could translate into delayed toxin clearance, hence the increased mortality of STEC-HUS in elderly patients [113]. Accordingly, immunodeficient mice develop renal and immunological manifestations comparable to HUS after inoculation with Stx, whereas wild mice are resistant [114,115].

## 6. Clinical Presentation

First, it should be kept in mind that approximately 75% of individuals [31,70] exposed to STEC will remain free of any symptoms. Factors determining a predisposition for developing colitis and HUS are only partially known, but age is believed to play a major role [113], and patients older than 65 years are at higher risk of developing a disease during outbreaks [11,37,116].

In children, STEC-HUS has a typical presentation with bloody diarrhea at the forefront. Symptoms develop after a median of four (1–10) days [33,117] and usually begin with abdominal pain and watery diarrhea, which may morph into bloody diarrhea only at a later stage.

Overt HUS develops in around 10% of symptomatic patients [67,118] (11% with O157:H7 infections versus 1% with non-O157 serogroups [25]) Acute kidney injury (AKI) requiring renal replacement therapy affects 30 to 61% [118,119,120] of pediatric patients. Proteinuria, leucocyturia and hematuria have also been described in 30%, 7% and 26% of patients, respectively [121]. Elevated blood pressure is reported in 15% of children [119] and in more than half of adults [122]. Histopathologic examination typically shows arteriolar and glomerular thrombosis, endothelial swelling with a widened subendothelial space [123], findings similar to other TMAs [124], although usually limited to the kidney [125]. Attempts to unravel a histological correlate specific of typical HUS have remained unsuccessful.

Extra-renal manifestations of STEC-HUS are less frequent but dictate the prognosis in children. Neurological manifestations occur in 20 to 25% [119] of children and can include seizures, coma, and paresis [126]. Cardiac involvement, although rare (<10% of STEC-HUS), is a cause of severe disease and death [127,128,129]. Although STEC were usually not considered as invasive pathogens, emergence of non-O157 serotypes have recently challenged this assumption: infections with O104:H4 or O80 serotypes may result in bacterial translocation and sepsis. These unusual clinical presentations are due to the acquisition of extra-intestinal virulence factors, such as the s88 plasmid for STEC O80 [130], or enteroaggregative features for O104:H4 [131]. Lastly, an increased risk of diabetes has been shown in survivors of STEC-HUS, which may be secondary to pancreatitis and ischemic damage of the islets of Langerhans during the acute phase [132,133].

### Clinical Specificities of Adult Patients: Not So Typical after All

In adults, the early etiological diagnosis of TMAs is intricate due to the wide range of competing diagnoses with a significant overlap of clinical and laboratory pictures [5,6]. Delineating STEC-HUS and other TMAs can be challenging (Table 3). The dearth of data related to the specific clinical features of STEC-HUS in adults represents another obstacle for elaborating criteria dedicated to on-the-spot recognition of STEC HUS.

In our experience, nearly 30% of STEC HUS adults had an underlying immunodeficiency [65]. Moreover, one or multiple conditions possibly contributing to the occurrence of a TMA are observed in one third of patients [65].

During the O104:H4 outbreak in Germany, the attack rate of bloody diarrhea among people who consumed contaminated sprouts was 27% [12], while the proportion of STEC-HUS among cases of STEC infections in adults culminated at 20% [14]. Besides, significantly reduced incubation periods were reported in older patients [77].

Extra digestive sources of STEC infection, such as the urinary tract, have been reported to trigger HUS in adults [134,135].

Severe neurological abnormalities are prevalent in adults, reaching two thirds of the patients from the Oklahoma registry [53], and half of the patients from both the O104:H4 outbreak [136] and the French National Reference Center (NRC) for TMA adults cohort [65]. Prominent manifestations were confusion or cognitive impairment (33.3%, 67.3% and 56.3%, respectively, in the Oklahoma registry, the German O104:H4 outbreak and the French registry), seizures (61.9%, 34.6% and 31.3%, respectively), and coma (38.1% and 37.5%, respectively, in the Oklahoma and French registries). In addition, neuropsychiatric symptoms are common in older patients [137]. These findings are in agreement with the results derived from the O104:H4 outbreak in Germany in 2011, in which the prevalence of neurological symptoms was similarly high [138].

Severe AKI is likewise a major concern in the adult population: replacement renal therapy was introduced in 42.9% of patients in the Oklahoma registry, and in 63.5% of the French national adult cohort, compared to 32.0% in the pediatric cohort in the same period in France.

## 7. Prognosis and Long-Term Outcomes

While two thirds of enterohemorrhagic *E. coli* (EHEC)-infected patients fully recover, identification of the risk factors predicting a fatal outcome or long-term sequelae is of critical importance. STEC-HUS is associated with a relatively low fatality rate in children, under 3% [53,76,120,139,140]. Mortality in the adult population offers a stark contrast, rising up to 15 to 33% in elderly and frail populations [11,37,40,53,61]. Long-term sequelae have been increasingly recognized in both populations, affecting about one-third of pediatric patients [141] and possibly more than 50% of adults [142].

### 7.1. Long-Term Complications of STEC-HUS in Adults

The kidney function and the central nervous system share the bulk of the burden with respect to long-term sequelae of STEC-HUS in children and in adults.

Long-term renal sequelae are a major fear of STEC-HUS both in children and adults [65]. Chronic kidney disease (CKD) requiring dialysis at discharge has been reported to be as high as 26% of patients alive at discharge in an elderly cohort [61]. In our experience, ongoing dialysis at discharge was required for 14% of survivors who required dialysis at the acute phase of STEC-HUS [65]. In a monocentric cohort study including adults from the 2011 O104:H4 outbreak, reduced estimated glomerular filtration rate <90 mL/min, de novo hypertension, and proteinuria (>30 mg albumin/L urin) were observed at one year in 47%, 25%, and 27% of the patients, respectively [143]. STEC-HUS has also been described as a cause of loss of kidney graft in adult transplant recipients [65,144]. The pathophysiology of CKD after STEC-HUS is primarily the end result of renal scarring in the form of glomerulosclerosis and tubulo-interstitial fibrosis. In turn, the reduced pool of nephrons induces hyperfiltration which may elicit the development of focal segmental glomerulosclerosis and hyalinosis, further aggravating the renal prognosis [145,146]. This finding is common to all AKI regardless of the cause [147], and it is reasonable to assume that the risk of CKD after a STEC-HUS episode in adults is not specific to this disease. However, studies comparing progression to CKD after STEC-HUS and other causes of AKI are currently lacking, especially in adults [148]. Moreover, CKD can emerge after a period of seemingly clinical and biological recovery in children [149]. At any rate, these results stress the need for a long-term follow-up of STEC-HUS patients, including in cases of apparent full recovery [121,149].

In children with STEC-HUS, intellectual performance does not seem to be impaired [150,151] even if subtle motoneuron impairment has been described, manifesting as poorer performances for both fine and gross movements and static balance, regardless of documented acute central nervous system involvement [151]. Results in adults show striking differences: neuropsychological symptoms, albeit minor, such as fatigue, headache, and attention deficit were present in 70% of adult patients 19 months after the O104:H4 outbreak. About 60% displayed abnormal neuropsychological assessment and 25% exhibited a cognitive decline [142]. Considering the severity of neurological manifestations in the acute phase of STEC-HUS, cerebral prognosis seems to be surprisingly good in adults from the O104:H4 outbreak of 2011, judging from long-term clinical and MRI evaluation of patients [136,142]. In contrast, chronic severe neurological impairment has been described in the case of cerebral hemorrhage and stroke [41,103].

Long-term gastrointestinal complications, such as colonic stricture, have also been anecdotally described as late complications of STEC-HUS [152]. Severe colitis can lead to abdominal surgery and colectomy [61].

As a consequence of the ischemic damage to the islet of Langerhans, delayed onset and recurrence of diabetes several years after the infection warrants long-term screening of STEC-HUS survivors [132,133,153].

Overall, the impact of STEC-HUS on the natural history of a preexisting condition is a major concern in elderly and frail patients [53,61].

### 7.2. Cause of Deaths of STEC-HUS in Adults

The main causes of death in STEC-HUS include direct complications of TMA, such as cerebral hemorrhage, ischemic stroke, cardiac involvement and encephalopathy, along with intensive care complications such as sepsis, ventilator-associated pneumonia, decompensation of an underlying comorbidity and limitation of care or refusal of dialysis, especially in the elderly [53,61,77,120].

### 7.3. Predictors of Long-Term Sequelae and Death

If the incidence and lethality of STEC-HUS is greater in young children (especially those under five years of age), the mortality and complication rate increase symmetrically with age in the adult population, with a dramatic rise in the over 60s [11,53,58,61,65]. The mortality rate in adults infected with STEC stood at 17.9% in the FoodNet registry cohort (USA, 2000 to 2006) [11], 33.3% in the Oklahoma registry (1989 to 2006) [53], and 19.8% in the French NRC for TMA adult cohort (2009 to 2017) [65]. By contrast, the lethality rate in pediatric cohorts is much lower (~3% and 0.9% in the USA [53,120] and French pediatric cohorts [76], respectively, over the same period). Patients over 60 years of age make up the majority of STEC-HUS-related deaths described in surveillance networks [11,53,58,78] (Table 2), and a similar observation can be inferred from outbreak-based registries [13,14,37,38,77] (Table 1). Patients older than 60 years are also more likely to be hospitalized during STEC infection [63], and to exhibit long-term complications in the case of STEC-HUS [53,61].

Comorbidities impact the likelihood of severe STEC-HUS in adults, and an underlying immunodeficiency has been associated with increased risk of death in STEC-HUS adult patients [61,65].

Akin to TTP or atypical HUS, unusual neurological symptoms at presentation are commonplace, causing diagnostic delays and impeding appropriate therapy [154,155]. In elderly patients, neurocognitive impairment appears to be a clinical prognostic marker of unfavorable outcome [37,65,154]. Severe neurological complications, including strokes, cerebral hemorrhage, and seizures, impact the vital prognosis [49,65,120,126,156], and are more frequent in adults, affecting up to two thirds of patients [53,60,61].

The time that elapses between inaugural symptoms and hospitalization is predictive of death [120,157]. Other clinical features associated with death in pediatric series include dehydration, central nervous system involvement, higher hematocrit, and high white blood cell and neutrophil counts, whereas dialysis requirement does not seem to be associated with death [37,120]. Data are scarcer in adults: in contrast to the pediatric setting, whereby the biological markers already described discriminate patients on admission according to prognosis, risk assessment in adult patients relies heavily on clinical events. Renal function at admission [158], neurological symptoms [153] and a higher hematocrit level [37] have been associated with a poorer prognosis. Cardiac involvement has been recognized as a harbinger of poor outcome in other forms of adult TMA, and troponin has been considered as a prognostic marker [154]. Most of these factors are common predictors of death in TMA from various etiologies [154,159,160], and they should be monitored to guide the care of patients.

## 8. Management

Important milestones have been achieved in understanding the pathophysiology of STEC-HUS. This progress has yet to translate into a specific treatment. Nevertheless, several ongoing studies are exploring new therapeutic avenues, and the lack of specific treatment should not divert us from non-specific measures which, especially in the most severe cases, remain essential.

### 8.1. Diagnosis

STEC screening is recommended in all patients presenting with HUS [7]. Stool STEC excretion in infected patients generally lasts less than a week following diarrhea occurrence [161]. Hence, the confirmation of STEC infection upon stool microbiological examination in the context of HUS is a time sensitive issue and stool sampling is therefore best collected within a six-day period following the onset of symptoms [74]. If stools cannot be collected, a rectal swab should instead be performed.

Stx genes and other virulence genes (*eae* and *ehxA*) are evidenced directly on stools by molecular methods following enrichment. Real-time PCR facilitates an expeditious diagnosis and is more sensitive compared to traditional methods; moreover, it remains positive for a median of 20 days [162]. STEC strains can be isolated by plating of fresh feces on selective and differential media [163].

Early isolation and characterization of STEC strains enable epidemiological surveillance and detection of clusters by performing molecular analysis, particularly via whole genome sequencing (WGS). Together with epidemiological data, these techniques have emerged as the most valuable tool for real-time discrimination between sporadic cases and outbreaks [164].

### 8.2. Preventive Measures

At the individual level, and as for all enteric infections, proper hand and food hygiene [165] are of the utmost importance. Vaccines have not proven their efficacy in humans [166], unlike those for cattle [167]. Farmers also play an important role in primary prevention, and several veterinary, dietary and farm practices must be implemented to prevent asymptomatic carriage of EHEC in cattle (fecal prevalence of *E. coli* O157 has been reported to be as high as 5 to 10% in adult beef [168]) to translate into human outbreaks [169]. Secondary prevention includes rapid notification to local public health authorities, source-control measures and isolation of confirmed cases [170].

### 8.3. The Quest for Specific Treatments

#### 8.3.1. Antibiotic Therapy

Antibiotic therapy during EHEC infection has been a matter of debate over the last decades [80]. ß-lactams, fluoroquinolones and trimethoprim-sulfamethoxazole induce the expression of bacterial SOS-response genes in response to DNA damage, and in the case of EHEC infection, this overexpression is coupled with an increased Stx phage gene expression [171]. This finding is supported by evidence from animal models [172,173,174] and retrospective studies [81,82] and has spurred British and North American disease societies to caution against the use of antibiotics in EHEC infection and STEC-HUS [175,176]. In contrast, protein and cell wall synthesis inhibitors such as azithromycin and aminoglycosides have been associated with a protective effect against the development of HUS [81,82,83], and early fosfomycin [177,178] or azithromycin [179] treatment may be of value in preventing long-term carriage of EHEC. A prospective randomized trial evaluating azithromycin in pediatric patients is currently recruiting (NCT02336516). Despite these results having been known for many years, and even though EHEC infection had been confirmed in FoodNet sites, 82% of adult patients received antibiotic therapy (67% of whom received fluoroquinolones) compared to 44% of children (RR 0.48 (0.41–0.57)) [180].

#### 8.3.2. Plasma Exchange and Immunoadsorption

Two randomized controlled trials evaluated the role of plasma infusion in children in the 1980s [181,182] without providing convincing results. In adults, plasma exchange and immunoadsorption have been used during outbreaks with conflicting results [183,184,185,186]. Nonetheless, some experts currently suggest the use of plasma exchange in the most severely affected patients, especially those with severe neurological involvement [187,188].

#### 8.3.3. Complement Inhibition

Following the report of three children successfully treated with the monoclonal anti-C5 antibody eculizumab [189], several reports of its off-label use during the O104:H4 outbreak in 2011 reported positive results in 11 children with severe neurologic symptoms [190] and in eight adults [39]. A larger, more robust analysis of 193 patients from the German STEC-HUS registry showed no significant effect of plasma exchange together with eculizumab [186].

Since then, case series and case control studies yielding equivocal results [191], and two randomized controlled trials completed in 2012 (NCT01410916) and 2018 (NCT02205541) without any publication have cast doubts over the efficacy of complement inhibition in this indication.

#### 8.3.4. Other Specific Treatments

Steroids [192], antithrombotic therapies [193,194,195], oral binding agents [196] and injectable Stx competitive inhibitors and antibodies [197,198,199,200,201] have been tested in the past or are under evaluation (NCT04132375) but have not reached the stage of clinical validation. Manganese has also been suggested as a potential low-cost treatment [202], owing to its capacity to block Stx anterograde transport to the Golgi apparatus, but this hypothesis has not been tested in the clinical setting.

#### 8.3.5. Intensive Care Management

Although only a minority of patients infected with EHEC develop STEC-HUS, approximately half of these cases require renal replacement therapy, and a significant proportion will require admission to an intensive care unit (ICU).

We recently reported 12 sporadic STEC-HUS adult patients (median age 64 (IQR 50–72) years) amongst 236 TMAs admitted to the ICU [6]. As previously shown [138], the prevalence of neurological symptoms, especially confusion, was outstandingly high. Five patients (42%) required renal replacement therapy, nine (75%) and three (25%) red blood cell (RBC) or platelet transfusions, respectively, nine were prescribed anti-hypertensive medications and two (17%) required mechanical ventilation. One (8%) patient died in the ICU.

In this section, we will describe nonspecific therapies required in STEC-HUS patients, with a focus on adult patients and on critical care management.

#### 8.3.6. Septic Shock

EHEC are not enteroinvasive pathogens [24] and sepsis is unusual in STEC-HUS patients. However, severe cases of hemorrhagic colitis can require surgery [203] and EHEC infections may occasionally manifest as sepsis or septic shock [204]. In particular, patients infected with *E. coli* from the O80 serogroup more frequently experience bacteremia and extra-intestinal infections [90,205,206].

Although critical care management of these patients should not differ from general guidelines in terms of blood pressure target, fluid resuscitation, and administration of antibiotic therapy within one hour after the recognition of sepsis [207], the need for bactericidal antimicrobial agents should be balanced against the risk of inducing Stx release with SOS-inducing agents [171].

#### 8.3.7. Fluid Administration and Hydroelectrolytic Balance

Timely fluid administration has been associated with a lower risk of developing HUS in children with EHEC infection [208,209,210,211]. This action is currently evaluated in a prospective trial (NCT03275792). In critically ill adult patients in general [212,213], and in those under renal replacement therapy specifically [214,215], fluid balance has been shown to influence survival. Altogether, these results call for increased alertness regarding fluid resuscitation in critically ill adults with STEC-HUS. The choice between saline and balanced crystalloids [216,217] has not been addressed in this indication.

#### 8.3.8. Blood Pressure Control

Severe hypertension can be a cause of TMA per se [218]; it may also aggravate endothelial injury in HUS and TTP [106,219,220]. In our experience [122], half of STEC-HUS adult patients present with hypertension and 79% require anti-hypertensive medications during their hospital stay. Unlike TTP, in which angiotensin-converting enzyme (ACE) inhibitors were associated with lower in-hospital mortality, we could not demonstrate any impact from the choice of anti-hypertensive drug used in STEC-HUS patients, but the long-term use of ACE inhibitors has been associated with a reno-protective effect [221].

#### 8.3.9. Transfusion

Ninety-three percent of STEC-HUS-infected children require RBC transfusion during hospital stay [120], compared to approximately 80% of adult patients [6,122], in which a mean of eight packs are transfused. The need for RBC transfusion seems to correlate with renal severity [222]. In other settings, investigators have worried about an increase in platelet clumping as a result of RBC transfusion, a scenario that has yet to be substantiated in STEC-HUS patients [223]. The implementation of early erythropoietin therapy has been devised as a means to reduce the number of RBC transfusions, yielding conflicting results [224,225], and is currently evaluated in a randomized controlled trial (NCT03776851). In patients affected with TTP [226,227] and in heparin-induced thrombocytopenia [228], platelet transfusions fuel the formation of microthrombi and thereby the microangiopathic process. In STEC-HUS, two case series failed to demonstrate such a risk [229,230]. However, given the low risk of hemorrhage observed in these patients [231] and the risk of allo-immunization, platelet transfusions should be restricted to bleeding complications or the need for invasive procedures (other than catheter placement and removal).

#### 8.3.10. Renal Replacement Therapy

Half of children [119,120] and an equal proportion of adults [6] with STEC-HUS will require renal replacement therapy during the acute phase. In adults, as opposed to children [232], peritoneal dialysis is rarely used in the ICU, and the literature concerning the benefits of continuous over intermittent hemodialysis is controversial. Therefore, the choice of renal replacement modality is mostly based on patients’ hemodynamic status, local expertise, and the availability of staff and resources [233].

Randomized trials have demonstrated [234,235,236] that delayed initiation of renal replacement therapy was a safe strategy to abrogate the need for dialysis in 30 to 40% of patients [236,237]. In the absence of specific data on the timing of renal replacement therapy in STEC-HUS patients, a delayed strategy seems to be most appropriate. Regional citrate anticoagulation [238,239] may prove to be useful in the case of hemorrhagic complications or severe thrombocytopenia.

## 9. Conclusions

STEC-HUS remains a major public health issue resulting from multiple routes of transmission and numerous bacterial strains. As compared to pediatric patients, adult STEC-HUS has distinctive features. First, the repertoire of alternative causes of TMA is far more diverse, thus posing consequential diagnostic challenges. A delayed diagnosis translates to a detrimental impact on the outcome. Second, the overall prognosis is bleak and even more so in elderly and frail patients. Chronic conditions, including immunosuppression and the occurrence of neurologic complications, predict poor outcome. Pending a significant therapeutic breakthrough, management remains hitherto largely supportive. Nonetheless, adult STEC-HUS cases should benefit from an interdisciplinary approach involving microbiologists, critical care specialists, and experts from various fields.

## Figures and Tables

**Table 1:** Comparison between children and adults in some of the main outbreaks of STEC-HUS from 1984 to 2015.

Year and Location	SeroType	STEC Infections N.		Including STEC-HUS Cases N. (%)		Deaths N. (%)	
		Children	Adults	Children ^1^	Adults ^2^	Children ^1^	Adults ^2^
1984, USA, Nebraska [46]	O157:H7	0	34	-	1 (2.94)	-	4 (11.76)
1985, Canada, Ontario [31]	O157:H7	0	73	-	12 (16.44)	-	17 (23.29)
1992, USA, Washington [32]	O157:H7	376	125	45 (9.00) (age range 1–68 years)		3 (0.60) deaths	
1990, Japan, Saitama [47]	O157:H7	121	0	14 (11.57)	-	2 (1.65)	-
1996, Japan, Sakai and Kyoto [33,34,35]	O157:H7	12680	47	121 (0.95)	3 (6.38)	3 (0.02)	1 (2.13)
1996, Scotland, Lanarkshire [36,37]	O157:H7	72	207	6 (8.33)	28 (13.53)	0 (0.00)	16 (7.73)
1999, China, Jiangsiu [38]	O157:H7	NA	NA	2 (1.03) ^3^	193 (98.97) ^3^	0 (0.00) ^4^	177 (91.71) ^5^
2006, USA, Utah and New Mexico [48]	O157:H7	10	13	7 (29.00) (age range 2–60 years)		0 (0.00)	0 (0.00)
2011, Europe, Germany [14]	O104:H4	NA	NA	101 (11.00) ^3^	744 (88.00) ^3^	1 (0.99) ^4^	53 (7.1) ^5^
2011, Japan, Toyama [49]	O111	41	45	23 (56.10)	11 (24.44)	3 (7.32)	2 (4.44)
2014, England, Dorset [42]	O55:H11	15	6	11 (73.00)	2 (33.00)	0 (0.00)	0 (0.00)
Total of Patients		13315	550	278	801	18	270
Overall proportion (random effect model)				17% (2–67)	15% (10–22)	1% (0–10)	9% (4–17)

^1^ Percentage among cases of STEC infection of children if not otherwise specified. ^2^ Percentage among cases of STEC infection of adults if not otherwise specified. ^3^ Percentage among total cases of STEC-HUS (845 STEC-HUS and 3816 STEC infections during the O104:H4 outbreak of 2011). ^4^ Percentage among cases of children with STEC-HUS. ^5^ Percentage among cases of adults with STEC-HUS. N.: Number. NA: Not available. HUS: Hemolytic and Uremic Syndrome. STEC: Shiga toxin producing *Escherichia coli*.

**Table 2:** Distribution of children and adults in some of the main surveillance network studies of STEC-HUS that included adults from 1984 to 2019.

Year and Location	Serotype	STEC Infections N. (%)		Including STEC-HUS Cases N. (%)		Deaths N. (%)	
		Children	Adults	Children ^1^	Adults ^2^	Children ^1^	Adults ^2^
1979–1983 Washington and Baltimore [60]	O157:H7	-	-	20	0	0	-
1990–1998 Wales [58]	O157:H7	204 (49.16)	211 (50.84)	17 STEC-HUS, age range 1–50		0 (0.00)	1 (0.47)
2000–2006 USA (8 states) [61]	O157:H7	1843 (55.50)	1478 (44.50)	190 (10.31)	28 (1.89)	8 (0.43) ^3^	13 (0.88) ^3^
1992–2012 Norway [28]	All	171 (51.35)	162 (48.65)	24 (14.04)	1 (0.62)	-	-
2017 Switzerland [62]	All	31 (25.83)	89 (74.17)	4 (12.90)	3 (3.37)	-	-
1989–2006 Oklahoma [53]	O157:H7	-	-	-	21	-	7 (33.33) ^4^
2009–2016 Alberta, Canada [63]	All	305 (44.59)	379 (55.41)	33 (10.82)	6 (1.58)	-	-
2009–2013 England [64]	O157:H7	1185 (36.56)	2056 (63.44)	66 (5.57)	20 (0.97)	-	-
2009–2017 France [65]	All	-	-	-	96	-	19 (19.79) ^4^
2014 USA (10 States) [66]	All	686 (53.93)	586 (46.07)	42 (6.12)	0 (0.00)	0 (0.00)	4 (0.58)
Overall proportion (random effect model)				9% (7–12)	1% (1–2)	0% (0–1)	1% (1–1)

^1^ Percentage among cases of STEC infection of children if not otherwise specified. ^2^ Percentage among cases of STEC infection of adults if not otherwise specified. ^3^ Including 5/15 deaths (33.3%) among patients > 60 years of age with STEC-HUS, 5/190 deaths among children (2.63%), and no death among the 13 adults of 18 to 59 years. ^4^ Percentage among cases of adults with STEC-HUS. N.: number. NA: not available. HUS: hemolytic and uremic syndrome. STEC: Shiga toxin producing *Escherichia coli*.

**Table 3:** Discriminative features between thrombotic microangiopathies: Shiga toxin *E. coli*-associated hemolytic uremic syndrome (STEC-HUS), atypical hemolytic uremic syndrome (aHUS), thrombotic thrombocytopenic purpura (TTP) and hypertension-related thrombotic microangiopathy (HT-TMA).

	STEC-HUS	aHUS	TTP	HT-TMA
Cause				
	EHEC	Deficiency or dysfunction of complement regulatory proteins	Deficiency or inhibition of the vWF-cleaving protease ADAMTS13	Malignant hypertension
Clinical Features				
Age	Young children (<five years) and older (>60 years) adults	Children and young adults	Middle-aged adults	Middle-aged adults
Sex ratio	Non-discriminant	Non-discriminant	Females > males	Males > females
Medical history	Not specific	Not specific	History of auto-immune diseases	History of hypertension
Blood pressure	Normal/moderate hypertension	Moderate/severe hypertension	Normal	Severe hypertension
Neurological symptoms	Confusion frequent in adults	Rare	Frequent, focal deficit	Headache
Digestive symptoms	Frequent but not systematic at the time of HUS, bloody diarrhea	Digestive symptoms frequent, diarrhea becomes rarely bloody	Rare	Rare
Biological Features				
Thrombocytopenia	Moderate	Moderate	Severe (<30 × 10^9^/L)	Inconstant to moderate
Acute kidney injury	Moderate to severe	Severe	Absent to severe	Severe
Specific Treatment				
	None	Eculizumab	Rituximab Caplacizumab	Blood pressure control

EHEC: enterohemorrhagic *Escherichia coli*, vWF: von Willebrand factor, ADAMTS13: A disintegrin and metalloprotease with thrombospondin type 1 repeats.

## Data Availability

Not applicable.
